# Supplementary material for: Late Split-Application with Reduced Nitrogen Fertilizer Increases Yield by Mediating Source–Sink Relations during the Grain Filling Stage in Summer Maize
Source: Plants (Basel). 2023 Jan 31;12(3):625. doi: 10.3390/plants12030625 (PMC9920228; doi:10.3390/plants12030625)
Supplement: Supplementary file 1 [file plants-12-00625-s001.zip › plants-2158314-supplementary.pdf]

Table S1. Average maize yields and N inputs since 2015 to 2018 at the NCP experimental site (located at Hebei, Shandong, Henan, and Shanxi provinces, China) based on published articles [8,54–63].

Region	Samples	N input (kg ha^{-1})	Yield (t ha^{-1})	Ears Per m^2	Kernels Per ear	100-kernels Weight (g)
Hebei	48	246±3	10.5±0.1	6.9±0.1	401±21	34.6±0.9
Shandong	16	224±7	9.3±0.2	6.7±0.9	514±11	33.7±1.5
Henan &Shanxi	4	223±8	9.7±0.2	6.7±0	527±17	31.7±0.4
Total	68	240±3	10.1±0.1	6.9±0.1	480±14	33.8±1.0

Samples=68.

References:

54. Li X.L., Guo L.G., Zhou B.Y., Tang X.M., Chen C.C., Zhang L., Zhang S.Y., Li C.F., Xiao K., Dong W.X., Yin B.Z., Zhang Y.C. Characterization of low-N responses in maize (*Zea mays* L.) cultivars with contrasting nitrogen use efficiency in the North China Plain. J. Integr. Agr., 2019, 18(9): 2141–2152.
55. Zhai L.C., Xu P., Zhang Z.B., Wei B.H., Jia X.L., Zhang L.H. Improvements in grain yield and nitrogen use efficiency of summer maize by optimizing tillage practice and nitrogen application rate. Agron. J., 2019, 111:666–676.
56. Liu M., Liang Q., Ge J.Z., Zheng Z.G., Zhang Y., Ma Z.Q., Wu X.D., Yang Y.A., Hou H.P. Effects of nitrogen and density on summer maize yield and nitrogen use efficiency. Acta Agriculturae Boreali-Sinica, 2019, 34 (6): 153-159.
57. Zhang P.P., Zhu Y.P., Huang L., Yu T., Qiao J.F., Li C., Zhang M.W., Liu J.B. Effects of reducing nitrogen applications on the physiological characteristics after anthesis of summer maize. J. Maize Sci., 2020, 28(4): 137-145, 164.
58. Yang F., Yan Q.Y., Lu J.X., Li F., Wang M., Dong F. Effects of nitrogen application on summer maize yield, nutrient utilization efficiency and soil available nutrient residues. Acta Agriculturae Boreali-Sinica, 2017, 32(1): 171-178.
59. Zhang L., Wu W.M., Chen H., Chen H.J., Peng C., Wang S.J., Cao C.F. Effect of nitrogen management on dynamic changes of soil inorganic nitrogen, yield, nitrogen uptake and utilization of maize. Soil and Fertilizer Sciences in China, 2021, 4: 126-134.
60. Li G., Bai Y.L., Yang L.P., Lu Y.L., Wang L., Zhang J.J., Zhang Y.J. Effect of drip fertigation on summer maize in north china. Scientia Agricultura Sinica, 2019, 52(11): 1930-1941.
61. Li G.H., Liu J., Dong S.T., Liu P., Zhang J.W., Zhao B., Shi D.Y. Effects of close planting and nitrogen application rates on grain yield and nitrogen utilization efficiency of different density-tolerance maize hybrids. Scientia Agricultura Sinica, 2017, 50(12): 2247-2258.
62. Li S.D., Feng B., Han W., Li W.J., Li Z.X., Wang Z.S., Li H.W., Bi X.J., Si J.S., Zhang B. Effect of optimized nitrogen application on summer maize yield and water- nitrogen utilization. Shandong Agricultural Sciences, 2020, 52(8): 57-63.
63. Ren H., Han K., Liu Y.E., Zhao Y.L., Zhang L.H., He Q.J., Li Z.H., Zhang J.B., Liu P., Wang H.Z., Zhang J.W., Zhao B. Improving smallholder farmers' maize yields and economic benefits under sustainable crop intensification in the North China Plain. Sci. Total Environ., 2021, 763: 143035.
